# FPGA Implementation of Keyword Spotting System Using Depthwise Separable Binarized and Ternarized Neural Networks

**DOI:** 10.3390/s23125701

**Published:** 2023-06-19

**Authors:** Seongwoo Bae, Haechan Kim, Seongjoo Lee, Yunho Jung

**Affiliations:** 1 School of Electronics and Information Engineering, Korea Aerospace University, Goyang-si 10540, Republic of Korea; tjddn1997@kau.kr (S.B.); ft0241@kau.kr (H.K.); 2 Department of Semiconductor Systems Engineering, Sejong University, Seoul 05006, Republic of Korea; seongjoo@sejong.ac.kr; 3 Institute of Semiconductor and System IC, Sejong University, Seoul 05006, Republic of Korea; 4 Department of Convergence Engineering of Intelligent Drone, Sejong University, Seoul 05006, Republic of Korea; 5 Department of Smart Air Mobility, Korea Aerospace University, Goyang-si 10540, Republic of Korea

**Keywords:** binarized neural network, field-programmable gate array, keyword spotting, ternarized neural network

## Abstract

Keyword spotting (KWS) systems are used for human–machine communications in various applications. In many cases, KWS involves a combination of wake-up-word (WUW) recognition for device activation and voice command classification tasks. These tasks present a challenge for embedded systems due to the complexity of deep learning algorithms and the need for optimized networks for each application. In this paper, we propose a depthwise separable binarized/ternarized neural network (DS-BTNN) hardware accelerator capable of performing both WUW recognition and command classification on a single device. The design achieves significant area efficiency by redundantly utilizing bitwise operators in the computation of the binarized neural network (BNN) and ternary neural network (TNN). In a complementary metal-oxide semiconductor (CMOS) 40 nm process environment, the DS-BTNN accelerator demonstrated significant efficiency. Compared with a design approach where BNN and TNN were independently developed and subsequently integrated as two separate modules into the system, our method achieved a 49.3% area reduction while yielding an area of 0.558 mm${}^{2}$. The designed KWS system, which was implemented on a Xilinx UltraScale+ ZCU104 field-programmable gate array (FPGA) board, receives real-time data from the microphone, preprocesses them into a mel spectrogram, and uses this as input to the classifier. Depending on the order, the network operates as a BNN or a TNN for WUW recognition and command classification, respectively. Operating at 170 MHz, our system achieved 97.1% accuracy in BNN-based WUW recognition and 90.5% in TNN-based command classification.

## 1. Introduction

Human–machine interface technologies, such as virtual reality, augmented reality, automatic speech recognition (ASR), and gesture recognition, have been extensively developed to improve interactions between humans and machines. Central to these technologies are keyword spotting (KWS) systems, a pivotal aspect of voice recognition technology. KWS systems operate on the principle of detecting specific “keywords” or phrases in spoken language, facilitating a plethora of applications, such as wake-up-word (WUW) recognition for device activation and command classification networks. These systems have found a broad range of applications, from robot vacuum cleaners to wearable devices and smart speakers.

Critical performance metrics, such as low-latency response, enhanced privacy, and reduced bandwidth and energy usage, have motivated the implementation of KWS systems in embedded platforms. With the advent of powerful, energy-efficient processors and the rise of edge computing and the Internet of Things (IoT), implementing KWS systems in embedded platforms has become a feasible way to realize these benefits. The implementation of KWS systems on diverse platforms, such as microcontroller units (MCUs) [1,2,3], field-programmable gate arrays (FPGAs) [4], and application-specific integrated circuits (ASICs) [5,6,7,8,9,10], represents a rapidly evolving research area, with each platform offering unique advantages and challenges. This research trend reflects the increasing demand for more efficient, high-performance KWS systems in a variety of applications and environments.

To implement a high-performance KWS system, two aspects must be considered: high performance in diverse environments and real-time performance guarantee on hardware with limited resources. The first aspect requires the efficient and high-performance processing of various functions, such as machine activation with WUW recognition and command classification tasks, on a single device. The second aspect requires ensuring real-time performance and limited area/power consumption without server intervention. These two problems must be solved simultaneously, and various solutions have been proposed due to their trade-off relationship.

Recent studies [11,12,13,14,15] have demonstrated the efficiency of deep neural networks (DNNs) in terms of performance and power consumption, with convolutional neural networks (CNNs) being applied in many vision and audio systems. However, DNN algorithms are difficult to apply to resource-limited embedded platforms owing to their high computational complexity and memory requirements. This has motivated researchers to explore more efficient, lighter alternatives. For instance, ref. [14] successfully implemented hardware-based KWS inference using temporal convolutional networks, achieving real-time inference with high accuracy and low power consumption. In another significant development, ref. [15] proposed memory-optimized long short-term memory (LSTM) that notably curbs energy usage.

In voice recognition technologies, both neural networks for WUW recognition and command classification are required. In KWS systems, one approach to support this series of processes is to implement a network with high bit precision such that WUW recognition and command classification pass through the same network. However, this may lead to memory waste during WUW recognition, which requires relatively low complexity. Therefore, this paper adopted a method for designing and integrating separate quantized networks with optimal bit precision for each application.

Owing to its simple classification task of distinguishing a few target keywords from an “unknown” keyword, research on WUW recognition has focused on increasing the quantization strength of the network to maximize its power efficiency [5,6,7]. Song et al. [5] proposed binarized neural network (BNN)-based voice activity detection (VAD) and a binarized weight network (BWN)-based wake-up system. The model achieved 32× and 5× reductions in memory and latency, respectively, compared with graphics processing units when classifying two keywords. Shan et al. [6] proposed a KWS system that included mel-frequency cepstral coefficient (MFCC) preprocessing and a BNN accelerator. Despite its relatively complex network architecture, this model minimized the memory size using depthwise separable convolutional neural network (DS-CNN)-based BNN techniques and classified one or two keywords. By using bitwise operations in hardware implementation, the BNN has the advantage of low power consumption and area while achieving sufficient WUW recognition accuracy depending on the network configuration.

To improve the accuracy of classifying multiple commands, researchers have studied quantized networks with higher bit precision than BNNs [8,9,10]. Liu et al. [8] proposed a BWN model to classify 10 keywords. Their approach dynamically selects fast Fourier transform (FFT) and coordinate rotation digital computer (CORDIC) units with 16-bit or 12-bit data paths, depending on the signal-to-noise ratio (SNR) of the input signal, and decides whether to use rectangularly mapped mel-filter banks. The pipeline structure and binarized weights minimize the size of on-chip memory, while the self-adaptive approximate computing method reduces the average energy consumption of the entire system. Gong et al. [9] proposed a selective quantized CNN that adjusts the accuracy by quantizing the data and weights to 4/8 bits according to the SNR. This model performed the command classification of 10 keywords. To overcome the limited accuracy of a BNN while maintaining a small memory size, this paper proposes a ternarized neural network (TNN) that extends the range of weighted values. The TNN was optimized for parallel operations with bitwise operations and implemented with fewer resources with an operation flow similar to that of the BNN. The memory optimization of different networks is possible with mode switching between the TNN and BNN.

In this paper, we employ a BNN for WUW recognition and a TNN for command classification. In conjunction with a DS-CNN, the system operates with the minimum number of parameters necessary to achieve the requisite accuracy for each function. The application of DS-CNN techniques in embedded systems is illustrated in references [3,16]. In [3], the authors proposed a KWS system that utilizes quantization and a DS-CNN. Although their 8-bit quantization of weights and data resulted in high accuracy in relatively shallow networks, our method reduces on-chip memory usage by implementing 1-/2-bit quantization. Moreover, our design permits bit-precision selection, thereby optimizing parameters for various networks. Ref. [16] demonstrated the implementation of real-time object tracking on an FPGA using binarized DS-CNN techniques. Although binarization techniques are beneficial for some applications, they pose challenges when implementing complex networks. We address this constraint in our paper by introducing TNNs, thus increasing the bit precision of the weights to 2 bits.

This paper proposes a KWS system consisting of a mel-processing unit (MPU) and a neural network unit (NNU). The proposed MPU detects valid voice signals through sensors and converts them into mel spectrograms, while the proposed NNU performs WUW recognition and command classification. This paper achieves high area efficiency by combining a BNN and a TNN, leveraging the similarities in their operation flows. The depthwise separable binarized/ternarized neural network (DS-BTNN) included in the NNU can be configured for specific purposes, such as weight bit precision, padding presence, and kernel size. We implemented and validated our proposed KWS system on an FPGA platform due to its inherent advantages, such as programmability and flexibility. Although the basic implementation was performed on an FPGA, we utilized a complementary metal-oxide semiconductor (CMOS) 40 nm process environment to compare the design area with the ASIC-based implementations described in other papers.

The remainder of this paper is organized as follows: Section 2 explains the mel-processing, BTNN, and DS-CNN algorithms. Section 3 describes the proposed KWS system. Section 4 explains the proposed hardware architecture. Section 5 presents the implementation results. Section 6 provides a thorough discussion, highlighting the strengths of our work using a comparison with other existing KWS systems. Finally, Section 7 concludes the paper.

## 2. Background

### 2.1. Mel-Processing Algorithm

Four digital feature extraction techniques are primarily used for voice data: MFCC, linear prediction coding coefficients, perceptual linear prediction (PLP), and relative spectral analysis–PLP [17,18,19]. The advantages and disadvantages of these approaches were evaluated in an experimental comparative analysis in [20]. The experimental results showed that the MFCC technique had excellent robustness and low computational complexity, making it highly suitable for feature extraction from data containing background noise or having a low SNR. This study adopted the MFCC technique, which consisted of the following feature extraction processes: pre-emphasis, framing, windowing, short-time Fourier transform (STFT), mel filtering, logarithmic operation, and discrete cosine transform (DCT).

The STFT was used to overcome the limitations of the FFT. In STFT operations, the input signal is divided into successive frames and multiplied by a Hamming window function to minimize the signal discontinuities before performing the FFT. The frames resulting from the STFT contain frequency information. Because this frequency information changes through successive frames in the time domain, they also include time information. An image with two-dimensional information in the time-frequency domain is called a spectrogram. The STFT equation in the discrete domain is as shown in (1).

(1) $$Y\left[m,f\right]=\sum_{n=-\infty }^{\infty }x\left[n\right]w\left[n-m\right]e^{-j\frac{2\pi nk}{N}}$$

where x(t) is the input signal, ω is the window function, and *m* is the window delay time.

The mel-filtering operation results in data in the form of a mel spectrogram by mapping a mel-filter bank that captures the characteristic sensitivity of the human auditory system to low frequencies and its insensitivity to high frequencies. A logarithmic operation is applied to reduce the distribution range of the mel filters; finally, the MFCC is obtained with the DCT.

### 2.2. Binarized/Ternarized Neural Network

CNN models mainly consist of convolutional layers (CLs) and fully connected layers (FCLs). A CL is a learnable layer that directly performs convolution operations on the input data to extract features without loss. A FCL performs linear transformations on a one-dimensional vector with a weight matrix using the results generated in the CL as the input data to perform the classification of the final label. Pooling layers reduce the data size to decrease computational complexity and prevent overfitting. Activation layers play an essential role in solving complex problems by introducing nonlinear characteristics into the network. Batch normalization (BN) [21] maintains the input data distribution to increase the learning speed and prevents the gradient vanishing problem. CNNs have been widely used in various image classification challenges owing to their robustness against image distortions and variations.

In general, the values, weights, and biases used in actual computations in CNNs consist of floating-point data. Moreover, operations such as multiplication and division used in CLs, FCLs, and BN not only utilize a large amount of memory but also increase power consumption owing to their high computational complexity. Thus, the application of CNNs to resource-constrained embedded platforms has been a challenge, leading to the emergence of CNNs with various lightweight techniques. BNNs [22] are representative lightweight CNNs obtained by compressing CNN activation and weights into 1 and −1 values instead of using single-precision floating-point data. We simplified the multiply–accumulate operation, which was previously complex and required multiple cycles in CLs, by replacing it with a simple bitwise operation using 1-bit XNOR and popcount operations [23]. While BN in neural networks using single-precision floating-point data involves complex operations, a BNN simplifies this process by adding an offset to the resulting value. BN has four fixed parameters for network inference operations. Because σ is always a positive value, it can be expressed by Equations (2) and (3), depending on γ [24].

$$Binarize\left(BatchNorm_{\gamma ,\beta }\left(x\right)\right)=Binarize\left(\frac{\gamma }{\sqrt{\sigma_{B}^{2}}}\left(x-\mu_{B}+\frac{\sqrt{\sigma_{B}^{2}}}{\gamma }\right)\right)$$

(2) $$=Binarize\left(x-\mu_{B}+\frac{\sqrt{\sigma_{B}^{2}}}{\gamma }\right)=Binarize\left(x+offset\right),\;\left(\gamma \geq 0\right)$$

(3) $$=Binarize\left(-\left(x-\mu_{B}+\frac{\sqrt{\sigma_{B}^{2}}}{\gamma }\right)\right)=Binarize\left(-x-offset\right),\;\left(\gamma <0\right)$$

BNNs compress weights and input data into single bits to significantly reduce memory usage and perform hardware-optimized parallel operations using bitwise operations such as XNOR and popcount. However, there are limitations to using BNNs for complex networks, such as multi-keyword detection, owing to the decrease in accuracy caused by lightweight techniques. To address this issue, we propose a TNN that maintains the input data as binary while ternarizing the weights. The TNN has higher accuracy than the BNN owing to its higher bit precision; however, it can still use the bitwise operation method, and both networks have similar operational processes.

### 2.3. Depthwise Separable Convolutional Neural Network

**Figure 1:**
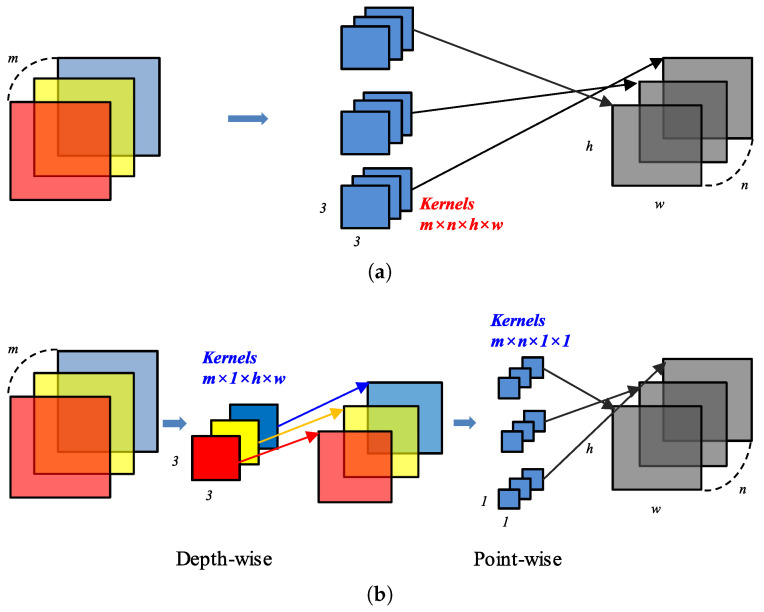
Comparison of CNN and DS-CNN: (**a**) CNN; (**b**) DS-CNN.

In a typical CNN, multiple three-dimensional kernels repeatedly multiply and accumulate input feature maps to generate multiple output feature maps, which is computationally intensive with large memory usage. To solve this problem, we applied a DS-CNN that is highly accurate compared with the same parameters while reducing memory usage. A DS-CNN performs the local and global feature extraction functions of a typical convolutional operation in separate layers. Depthwise (DW) convolution matches a single input channel to an output channel, excluding interchannel correlations and reflecting local features. Pointwise (PW) convolution is equivalent to 1 × 1 convolution, reflecting interchannel correlations (i.e., global features). Figure 1 shows CNN and DS-CNN. In this figure, the use of the same color (e.g., red, blue, yellow) represents input channels with the same index being used to generate corresponding output channels in DW convolution. Table 1 lists the number of parameters and computations in specific layers with a 3 × 3 kernel. In one example from the network used in this paper, a layer with 128 input channels and 64 output channels experienced an approximately eight-fold reduction in the number of parameters and computational complexity using the DS-CNN.

## 3. Proposed KWS System

**Figure 2:**
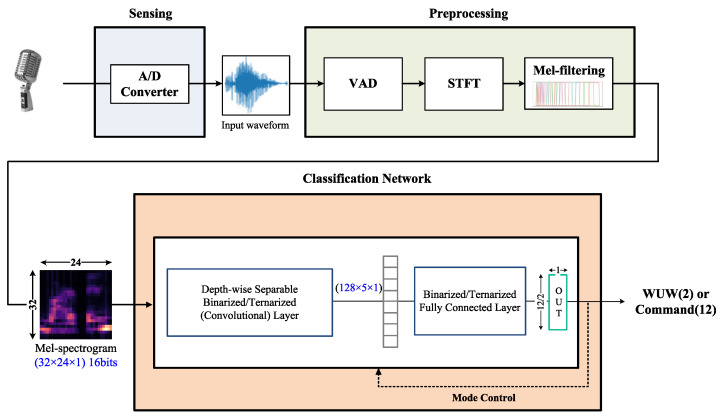
Overview of the proposed KWS system.

The proposed KWS system is shown in Figure 2. During the initial operation, energy-based VAD detects speech data and preprocesses them into a mel spectrogram, which is then sent to the network set in binary mode for WUW recognition. When the input speech data are recognized as a WUW, the network switches to ternary mode, and the next input data undergo the same preprocessing before being sent to the network with the revised settings for command classification. This enables voice identification, preprocessing, machine activation, and command classification functions to be performed on a single device. The target network, DS-BTNN, was designed to support various networks by allowing users to select configurations such as bit precision (1/2 bits), padding, BN, and kernel size. The input layer uses a binarized/ternarized weight network because mel spectrograms contain a large amount of information despite their small data size; therefore, only the weights are quantized instead of the input data.

### 3.1. Preprocessing

**Figure 3:**
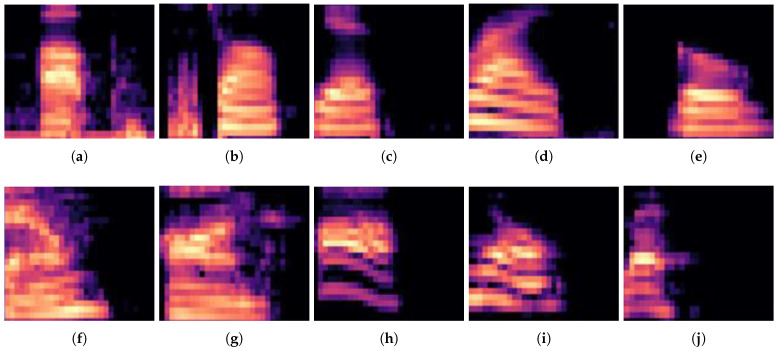
Mel-spectrogram data sample extracted from the Google Speech Commands Dataset: (**a**) up; (**b**) stop; (**c**) yes; (**d**) right; (**e**) go; (**f**) down; (**g**) on; (**h**) off; (**i**) no; (**j**) left.

The hardware resources used in this study were minimized by excluding windowing, log-scaling, DCT, and unnecessary operations from the general MFCC process without affecting network accuracy. Experiments confirmed that the accuracy of mel spectrograms, which visually distinguish keywords, was high when trained on datasets with little noise and few keywords. This was achieved by excluding the DCT, which is the last step in the MFCC process, and the mel spectrograms of the dataset used in the experiment, as shown in Figure 3.

**Figure 4:**
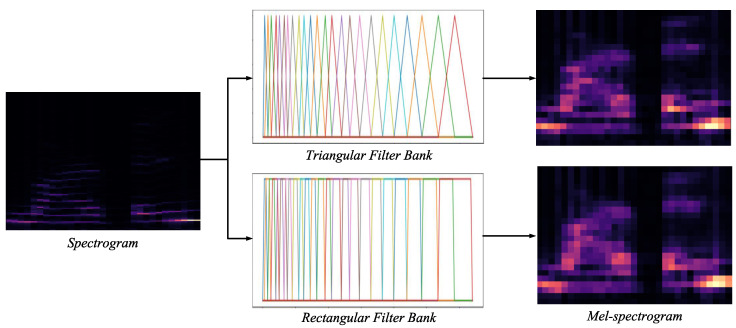
Difference between triangular/rectangular filter banks.

The speech signal had a sampling frequency of 8 kHz, using 4224 data points for 0.528 s. Each frame for the STFT calculations consisted of 256 data points. Because 50% of the overlapping frames were used, a 256-point FFT was performed each time 128 data points were inputted. Mel filtering proceeds by mapping the mel-filter bank, which contains the characteristics of the human auditory structure that are sensitive to low frequencies and insensitive to high frequencies. The mel-filter bank has a huge memory requirement during hardware operations because of the information on the coefficients of all filters. In this study, the positive values of the triangular filter were replaced with 1 and mapped to a rectangular filter, while the complex look-up table (LUT) and multiply–accumulate operations were replaced with simple cumulative sum operations. As seen in Figure 4, the results of the two filters are difficult to distinguish, and no performance degradation occurred during actual training. Different colors in the filterbank in figure are used solely for visual distinction among the filters.

### 3.2. Performance Evaluation with Network

The dataset adopted for the KWS system performance comparison was the Google Speech Commands Dataset (GSCD) [25], which contains over 105,000 voice data with a length of 1 s for 30 keywords. For comparison with reference studies, the following words were used in command classification: “up”, “stop”, “yes”, “right”, “go”, “down”, “on”, “off”, “no”, “left”, “silence”, and “unknown”. The command classification task consisted of 27,520 training data points for learning and 6880 test data points for validation. For WUW recognition, one keyword was reduced, while the others were treated as “unknown” after adjusting the ratio between data. The entire dataset consisted of 3840 training data and 960 testing data.

Pytorch [26], version 1.13.1, was used to train the network on the GSCD. Training was conducted using a cross-entropy loss function and the improved Adam optimizer [27] on NVIDIA GeForce RTX A6000 with CUDA version 12.1. The batch sizes were set to 256 and 128 for command classification and WUW recognition, respectively, with a total of 100 epochs. The learning rate was set to 0.005 for the first 40 epochs, 0.001 for the next 40 epochs, and 0.0005 for the remaining epochs. The network employed quantization techniques (BNN/TNN) and a DS-CNN. Experiments were conducted to analyze the changes in accuracy and parameters due to the number of nodes, the CL and FCL configurations, and the application of the DS-CNN technique using various networks. While the number of nodes was kept almost constant to satisfy the accuracy requirement, the number of parameters varied depending on the CL and FL configurations, and DS-CNN applications. The comparison results in Table 2 show that the proposed Network 4 achieved both high accuracy and an optimized number of parameters.

**Figure 5:**
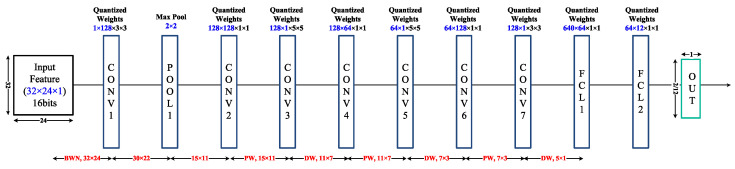
DS-BTNN network structure.

The final network structure proposed in this study, the DS-BTNN, is a combination of DS-CNN and BTNN. Its structure is shown in Figure 5. The entire network consists of nine layers (CL + (DW CL + PW CL) × 3 + FCL × 2), all of which enable BN and sign activation, except for the output layer. Max pooling was performed in the first layer.

## 4. Proposed HW Architecture

### 4.1. Mel-Processing Unit

**Figure 6:**
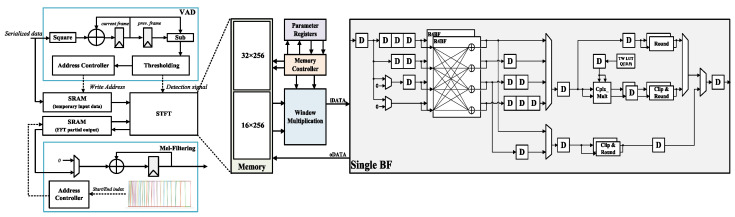
Hardware architecture of proposed mel-processing unit.

Figure 6 shows the proposed MPU, which consists of an energy-based VAD block that verifies the validity of the continuous data inputted through the Pmod terminals of the ZCU104 board, an STFT block for analyzing frequency changes over time in the continuous data, and a mel-filtering block that reflects the characteristics of the human auditory structure.

Energy-based VAD is a widely used algorithm for extracting actual voice intervals from continuous data. It signals the start of a valid voice signal when the difference between the energy levels in a specific section and in the previous section exceeds a certain threshold. The address controller within VAD stores the data with address values ranging from 0 to 127 for every 128 data inputs. After validation, it fills the data with address values between 128 and 255 to start the 256-point FFT. It performs a 50% overlapped 256-point FFT by storing values in the appropriate addresses for every subsequent 128 data inputs.

The STFT process is performed using the fixed-point radix-4 algorithm of the decimation-in-frequency method for every 256-point FFT computation. It takes serialized data as input, performs radix-4 operations using a buffer, and stores the partial product of the FFT in a 32 × 128 local memory. As the input data are in the real-number format, the FFT derives 129 non-overlapping complex numbers. Approximation techniques are used to replace complex calculations such as multiplication and squaring that are required to calculate the absolute value in mel filtering with simpler operations, such as bit shift and addition [28]. The equation is expressed as (2).

(4) $$∥x∥=\frac{3}{8}\left(\left|Re(x)\right|+\left|Im(x)\right|\right)+\frac{5}{8}max\left(\left|Re(x)\right|,\left|Im(x)\right|\right)$$

Subsequently, the spectrogram obtained from the STFT is passed through the mel-filter bank for filtering. By quantizing the triangular filter and converting it into a rectangular filter, filtering can be performed using simple accumulation operations. The LUT stores the start and end indices of the FFT results that correspond to each filter. Then, the data are read from the on-chip memory based on the index and accumulated. The resulting values are stored in the double data rate (DDR) memory and used as input data for the NNU.

### 4.2. Neural Network Unit

**Figure 7:**
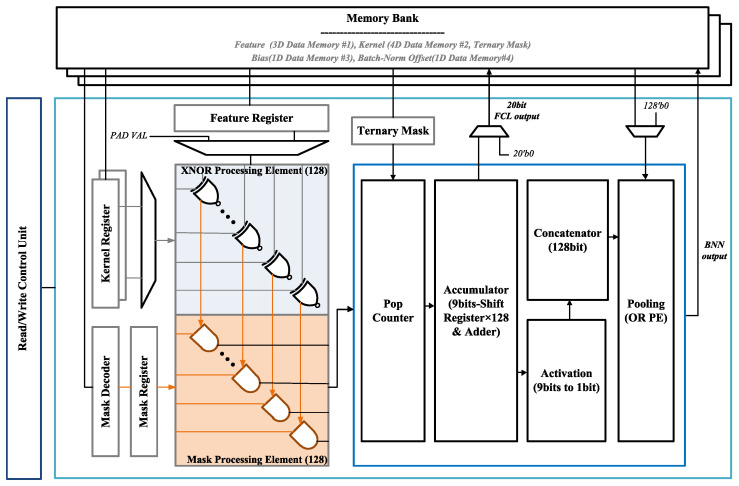
Hardware architecture of proposed neural network unit.

As shown in Figure 7, the proposed NNU consists of an XNOR processing element for storing the input data, two popcounters, an accumulator, an activation block, a concatenator, and a max-pooling block. Each block was designed with variable parameters to accommodate various network topologies. The NNU was designed as a layered accelerator structure that utilizes fewer resources. It is also reusable compared with the streaming accelerator structure, making flexible applications in various scenarios possible. In addition, the parallel computing structure that processes data on a channel-by-channel basis maximizes the acceleration effects and supports adaptive parallel computing for each layer, enabling optimized parallel processing in all layers.

The entire network system operates by repeating the DS-BTNN layers for a certain number of times. When each layer operation begins, the on-chip memory in the processor containing the NNU retrieves the data allocated to the layer from the external DDR memory. Therefore, the size of the on-chip memory is set according to the layer that uses the most parameters, which currently occupies 18.7 KB.

The NNU significantly reduces memory usage by binarizing the network input and ternarizing the weights according to the target. Convolution operations are replaced with XNOR and popcount operations, which significantly reduce the number of computations. It supports variable kernel sizes (1 × 1, 3 × 3, and 5 × 5), variable input/output channel sizes, and various operation modes, allowing not only standard CNNs but also depthwise convolution, pointwise convolution, and fully connected operations to be performed. In addition, each layer can freely set the quantization levels, padding, pooling, bias, and BN options.

The NNU operation is processed as follows: In a BNN, the input data and weights are strictly composed of only 1 and −1. This allows the XNOR operation to perform multiplication. Depending on the size of the input channel, designed hardware can perform XNOR operations on up to 128 bits of data. After the XNOR operation, the results must be accumulated. Since the result of the XNOR operation consists of 1 and −1, we utilize a popcounter that counts the number of 1 and −1 in 128-bit data instead of a traditional adder. This effectively replaces the accumulation sum, maximizing parallelism. In the case of the TNN, it can be computed in the same way, since it consists of −1, 0, and 1. The cumulative sum is passed through a sign activation block, where it is again binarized to have a value of 1 or −1. The concatenator bundles the binarized values into 128 bits at a time. The max-pooling block optionally reduces the size of the output data by a quarter. The NNU can work as an FCL for the final output by resizing the input data and omitting the activation block.

## 5. Implementation Results

**Figure 8:**
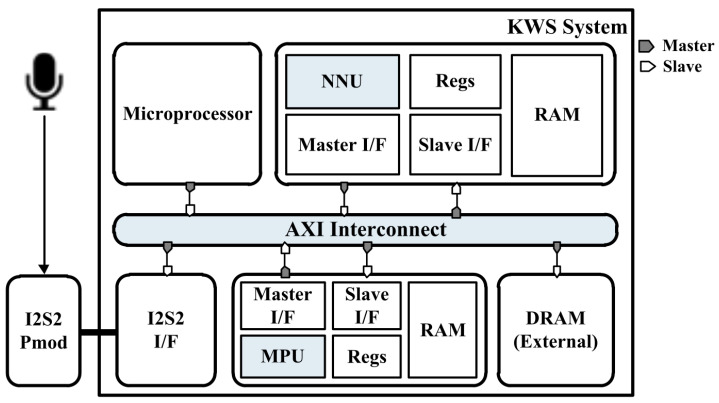
FPGA platform for the verification of the proposed KWS system.

The proposed KWS system is implemented on an FPGA platform with an advanced extensible interface (AXI) bus interface for verification. As shown in Figure 8, the FPGA platform consists of a KWS system that performs voice signal classification. This system includes two processors, each with an I2S2 interface, a microprocessor, a DDR memory, an NNU, and an MPU. Pmod I2S2 includes an analog-to-digital converter that converts the serial voice signal input from the microphone into 16-bit data and sends data bundles of a certain size to the MPU through the I2S2 interface. Each processor contains a DDR memory, an I2S2 interface, a master interface for communication with the processor, a slave interface for communication with the microprocessor, and on-chip memory for storing input/output data. The on-chip memory utilized in this study was BlockRAM with dual port as a hardware component. In addition, both processors have registers for the mode settings, including the quantization levels and BN. This system efficiently transfers data using a 64-bit AXI bus interface for high-bandwidth and real-time communication between the processor and other components.

In this design, the MPU and NNU were designed to operate independently. The data flow, based on the AXI4 bus system, is as follows: The microprocessor sends a start signal to VAD within the MPU to initiate voice signal detection. VAD detects the speech signal in the continuous data received from the microphone. Eventually, it processes the mel-spectrogram data and stores them in the DDR memory connected to the AXI4 bus system. Once storage is complete, the microprocessor receives the end signal from the MPU and sends the start signal to the NNU. Before starting the computation, the NNU reads the mel spectrogram and the parameters for the first layer from the DDR memory. The NNU passes through several layers and delivers the final result to the microprocessor, which then repeats the process of detecting speech using VAD. In this case, the NNU operates in BNN mode for the first speech signal, assuming it to be the WUW, and then processes subsequent input with the TNN for command classification.

**Figure 9:**
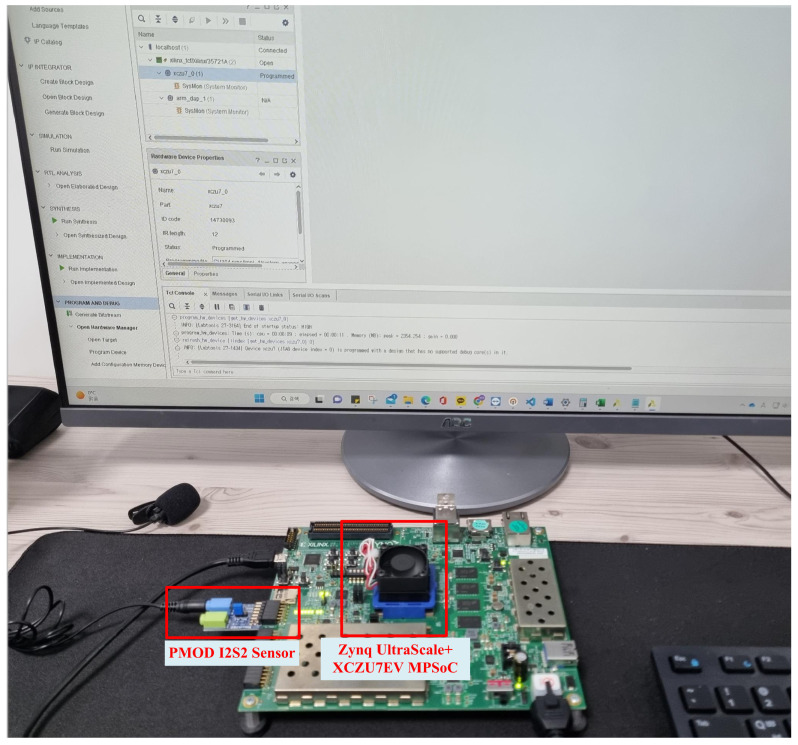
Verification environment using an FPGA platform.

The proposed KWS system was designed using the Verilog hardware description language at the register transfer level, and implemented and verified on the Xilinx UltraScale+ ZCU104 FPGA platform using the Vivado tool. The output results on each platform were compared and verified using the vectors in the Python-based simulator. Table 3 summarizes the resource consumption of the proposed KWS system. The proposed KWS system was synthesized using 27,315 configurable logic block (CLB) LUTs, 25,534 CLB registers, and 31 digital signal processors (DSPs). These values correspond to 11.7% of the total LUTs, 5.42% of the total registers, and 0.87% of the total DSPs on Ultrascale+ MPSOC with XCZU7EV-2FFVC1156. The synthesis results confirm that the system could operate at up to 170 MHz. Figure 9 shows the verification environment on the FPGA platform. The sensor connected to the Pmod terminal of the ZCU104 FPGA board shown in the figure is Pmod I2S2, which has analog-to-digital converter and digital-to-analog converter circuits connected to each of the two audio jacks. These circuits help to transmit and receive data via the I2S protocol, and in this design, a microphone was connected to the input terminal to receive real-time voice signals.

In this study, we utilized Synopsys tools to ascertain the area of the implemented design, thereby making comparisons with other studies that implemented ASICs possible. The RTL code was subjected to lint checking using the SpyGlass tool, and RTL simulation was executed using the VCS tool. In addition, by applying constraints related to the CMOS 40 nm process, we performed synthesis using the Design Compiler RTL synthesis solution, resulting in an area measurement of 0.558 mm${}^{2}$.

## 6. Discussion

In this chapter, we will provide detailed analysis and comparison of our findings in the broader environment of KWS systems. We will begin with a comparative analysis of our proposed system synthesized in a CMOS 40 nm process environment and other KWS systems, as presented in Table 4. In this comparison, we will focus on several key performance metrics, such as the number of target keywords, network accuracy, on-chip memory size, and normalized area. All the compared systems utilized the GSCD and were implemented in ASICs using different quantization strategies. After mentioning the implications and strengths of our results, we will discuss the limitations and future research directions in detail in the conclusion section.

In [6], a BNN was used to recognize one to two keywords. Although the memory size and area were small owing to the application of quantized networks and DS-CNN techniques, and a small number of keywords was supported, the number of detectable keywords could not be increased owing to factors such as the reduced accuracy of the BNN. In this study, a TNN, which has an operation flow similar to that of a BNN, was applied to achieve high accuracy and enable the recognition of 10 commands.

In [8,9,10], 10 keywords were classified, similarly to the proposed system. In [8], a full BWN structure with only weight binarization was used. By applying a pipeline structure instead of layer-by-layer operations, the on-chip memory size is minimized, and a concise network structure is achieved owing to the high bit precision of the data. However, the area is larger than that of the proposed processor. In [9], a system that adjusts the quantization bits of the input data and weights to 4/8 bits depending on the noise of the input data was proposed. Because up to 8-bit input data and weights are used for the same dataset, it has the advantage of accuracy. However, a relatively large amount of resources are utilized compared with the present study, with memory size and total chip area of 69 KB and 1.42 mm${}^{2}$, respectively. In addition, the system proposed this study can operate with minimum parameters by applying TNN for 10 keywords and partially applying BNN for 1–2 keywords.

Our study presents the hardware implementation of a KWS system that demonstrates significant improvements in area efficiency. We achieved the lowest normalized area compared with similar studies, while maintaining a high balance among network accuracy, on-chip memory size, and latency. This efficiency could open up new possibilities for integrating more advanced features in embedded systems with limited resources, thereby expanding the applications of KWS technologies.

## 7. Conclusions

In this paper, we propose a KWS system for real-time WUW recognition and command classification on a single device. To apply the complex CNN structure to the limited hardware structure, we used a quantization technique and a DS-CNN. In addition, we optimized memory usage by varying network components based on purpose.

In our experimental setup, we integrated a voice input sensor, an MPU, and an NNU. During the experiment, the system detected valid voice inputs, converted them into mel spectrograms, and then processed these through a BNN and a TNN for WUW recognition and command classification. The success of the preprocessing, WUW recognition, and command classification processes was confirmed by cross-verifying results between the Python and FPGA platforms. The proposed system demonstrated significant efficiency, achieving BNN-based WUW recognition and TNN-based command classification accuracy rates of 97.1% and 90.5%, respectively. Moreover, it showed effective resource management, with only 18.7 KB of on-chip memory usage and an area of 0.558 mm${}^{2}$ in a CMOS 40 nm process environment.

The study’s limitation lies in its inability to derive power consumption due to a lack of actual ASIC implementation. Future work should aim to quantify this parameter and further optimize the network architecture. Testing against a broader set of keywords and research on transformer and lightweighting techniques for different DNN architectures are also planned.

## Figures and Tables

**Table 1:** Comparison of the resource consumption of CNN and DS-CNN.

	Memory	Operation
CNN	*m* × (9 × *n*)	*h* × *w* × *m* × (9 × *n*)
DS-CNN	*m* × (9 + *n*)	*h* × *w* × *m* × (9 + *n*)

**Table 2:** Accuracy and parameters of DS-BTNN.

Network	Feature Extraction				Classification			Accuracy (%)	Parameter (KB)
	C ^1^1	C ^1^2	C ^1^3	C ^1^4	F ^2^1	F ^2^2	F ^2^3		
1	128	128	64	–	128	64	12	95.5	969,600
2	128	128	64	128	64	12	–	93.4	732,032
3	128	128 ^3^	64 ^3^	–	128	64	12	90.7	212,928
4	128	128 ^3^	64 ^3^	128 ^3^	64	12	–	90.4	83,264
5	64	64 ^3^	32 ^3^	64 ^3^	32	12	–	82.3	28,000

^1^ Convolution layer; ^2^ fully connected layer; ^3^ depthwise separable convolutional layer.

**Table 3:** Implementation results of the proposed KWS system.

Unit	CLB LUTs	CLB Registers	DSPs
MPU	5349	3735	5
NNU	11,270	9434	26
AXI Interconnect	10,696	12,365	–
Total	27,315	25,534	31

**Table 4:** Comparison results between the proposed KWS system and previous implementations.

Ref.	[10]	[6]	[8]	[9]	Proposed
Process	65 nm	28 nm	22 nm	22 nm	40 nm
Latency	16 ms	64 ms	16 ms	64 ms	22 ms
Normalized latency (40 nm)	10 ms	91 ms	29 ms	116 ms	22 ms
On-chip memory	65 KB	2 KB	11 KB	69 KB	18.7 KB
Network structure	LSTM	DS-BNN	BWN	QNN	DS-BTNN
Bit width (weight)	4/8 bits	1 bit	1 bit	4/8 bits	1/2 bits
Bit width (data)	10 bits	1 bit	16 bits	4/8 bits	1 bit
Keywords	10	1/2	10	10	1/10
Accuracy (%, 1 word)	-	98	-	-	97.1
Accuracy (%, 10 words)	90.87	-	87.9	88/93 (4/8 bits)	90.5
Area (mm${}^{2}$)	2.56	0.23	0.60	1.42	0.56
Normalized area (40 nm, mm${}^{2}$)	0.97	0.47	1.99	4.69	0.56

## Data Availability

Not applicable.
